# CircATRNL1 activates Smad4 signaling to inhibit angiogenesis and ovarian cancer metastasis via miR‐378

**DOI:** 10.1002/1878-0261.12893

**Published:** 2021-01-11

**Authors:** Juan Wang, Yan Li, Jin‐Hua Zhou, Fang‐Rong Shen, Xiu Shi, You‐Guo Chen

**Affiliations:** ^1^ Department of Gynecology and Obstetrics the First Affiliated Hospital of Soochow University Suzhou China; ^2^ Department of Gynecology and Obstetrics the First People's Hospital of Yancheng China

**Keywords:** angiogenesis, metastasis, ovarian cancer, Smad4 signaling

## Abstract

Ovarian cancer is one of the most frequent carcinomas in females, and the occurrence rate is still rising despite many advances made. The pathogenesis of ovarian cancer remains greatly unclear. Here, we investigated the mechanisms of ovarian cancer, with the focus on circATRNL1. Human ovarian cancer tissues and cell lines were used to examine levels of circATRNL1, miR‐378, Smad4, AKT, and other proliferation‐related and migration‐related proteins. Cellular assays were used to determine cancer cell proliferation, invasion, migration, apoptosis, and angiogenesis. We validated the interactions of circATRNL1/miR‐378 and miR‐378/Smad4, and a mouse tumor xenograft model was employed to assess the effect of circATRNL1 on tumor growth and metastasis *in vivo*. We found that circATRNL1 was decreased while miR‐378 was increased in human ovarian cancer tissues and cells. circATRNL1 bound to miR‐378 while miR‐378 directly targeted Smad4. Overexpression of circATRNL1 or knockdown of miR‐378 suppressed angiogenesis and ovarian cancer cell proliferation, invasion, and migration via decreasing proliferation‐ and migration‐related proteins via miR‐378 or Smad4, respectively. Overexpression of circATRNL1 restrained ovarian cancer growth and abdominal metastasis *in vivo*. Our findings indicate that circATRNL1 acts as a miR‐378 sponge to active Smad4 signaling and suppresses angiogenesis and ovarian cancer metastasis.

## Graphical abstract

Here, we report that the expression of circATRNL1 is low in ovarian cancer cells, leading to increased miR‐378 levels and reduced levels of the downstream target Smad4. Concomitantly, activated AKT signaling promoted cancer cell proliferation, angiogenesis, as well as metastasis.

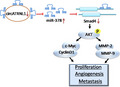

**Abbreviations**
circRNA: circular RNA
DMEM: Dulbecco's modified Eagle's medium
FISH: fluorescence in situ hybridization
HUVEC: human umbilical vein endothelial cell
IHC: immunohistochemistry
miRNA: microRNA
MMP‐2: matrix metalloproteinase‐2
MMP‐9: matrix metalloproteinase‐9
RIP: RNA immunoprecipitation
TGF‐β: transforming growth factor‐β
VEGF: vascular endothelial growth factor

## 1 Introduction

Ovarian cancer is one of the most frequent and lethal malignancies for women throughout the world with an estimated 152 000 death per year, and its occurrence rate is on a steady rise [1, 2, 3]. Due to its deep location in the pelvic cavity, ovarian cancer is hard to diagnose in early phases wherein symptoms are normally atypical or absent, and thus, the majority of patients are diagnosed at late stages wherein surgical treatments, chemotherapy, and radiotherapy are limited [4]. Further, ovarian cancer has high potential for metastasis and recurrence [5]. Consequently, the prognosis of the disease is still badly poor. Despite the advances in research, the etiology of ovarian cancer is very complex and still not fully understood. It is very necessary to explore the underlying mechanisms to advance the development of new effective therapy.

Circular RNAs (circRNAs) are a relatively new family of endogenous noncoding RNAs that are featured by the closed circular structures without the 5′ cap and 3′ poly adenylation tail [6, 7, 8]. They are very stable in cells. Although the functions of circRNAs remain largely unknown and lack a consensus, many studies have shown that their expressions are developmentally regulated, and tissue‐ and cell‐specific, and that they have important roles in cellular processes [6]. CircRNAs modulate gene expression through sponging endogenous RNAs like microRNAs, or interacting with RNA binding proteins [7]. Recently, accumulating studies show that circRNAs play crucial roles in cancer development and progression [7, 9]. Aberrant or dysregulated expressions of circRNAs have been reported in many cancers, such as hepatocellular cancer, gastric cancer, and lung cancer [7, 8, 9]. In ovarian cancer, emerging evidence has demonstrated that circRNAs are crucial regulators. For instance, circHIPK3 suppressed ovarian cancer progression while circGFRA1 promotes [10, 11]. However, the detailed picture remains incomplete. Recently, it has been reported that circATRNL1 (hsa_circ_0020093) is reduced in ovarian cancer [11]. circATRNL1 has been shown to promote oral squamous cell carcinoma radio‐sensitivity [12]. However, the function and underlying mechanism of circATRNL1 in ovarian cancer are not well studied and require further investigations.

microRNAs (miRNAs) are the well‐known family of endogenous noncoding RNAs being extensively characterized and play important roles in regulating gene expression [13, 14]. miRNAs are greatly involved in numerous cellular processes and varieties of diseases including cancers [15]. In ovarian cancer, many dysregulated miRNAs have been observed, such as miR‐145, miR‐199, and miR‐200 [16, 17, 18]. A previous study has profiled the differentially expressed miRNAs in ovarian cancer cells and miR‐378 was identified as one of them [19]. miR‐378 has been reported elevated in ovarian cancer, and it could serve as a biomarker for response to anti‐angiogenic treatment in ovarian cancer [20]. Nevertheless, its exact function lacks understanding. Many circRNAs are able to act as miRNA sponge and our bioinformatic analysis (Starbase) suggests that circATRNL1 might bind with miR‐378. Therefore, we aimed to investigate the potential function of circATRNL1/miR‐378 in ovarian cancer.

Smads are a family of proteins that transduce extracellular signaling to the nucleus and Smad4 is a critical member that is activated by transforming growth factor‐β (TGF‐β)‐related ligands [21, 22]. Smad4 plays essential roles in many cellular processes like cell proliferation, growth, differentiation, and migration [21]. Further, a great number of studies indicate that activation of Smad4 contributes to cancer development and progression [23, 24]. In the early phase, activation of Smad4 results in cell cycle arrest and apoptosis, suppressing cancer development [24]. Later it is involved in tumor angiogenesis and metastasis [24]. Loss of Smad4 plays a causal role in facilitating the initiation and development of varieties of cancers, such as colorectal carcinoma, squamous cell cancer, and pancreatic cancer [25, 26, 27]. For example, in colorectal cancer, knockdown or deletion of Smad4 promoted cancer migration and invasion via activating AKT signaling [27]. In ovarian cancer, Smad4 level was reduced in cancer cells and loss of Smad4 in blood vessel endothelial cells facilitated cancer metastasis [28]. Through bioinformatic analysis (Starbase), we found some complementary binding sequences between miR‐378 and Smad4 mRNA. We hypothesized that miR‐378 is involved in ovarian cancer through Smad4 signaling.

In this study, we fully investigated the role of circATRNL1/miR‐378/Smad4 axis in ovarian cancer. We found that circATRNL1 was reduced while miR‐378 was elevated in ovarian cancer tissues and cells. circATRNL1 bound with miR‐378 while miR‐378 targeted Smad4 mRNA. circATRNL1 suppressed ovarian cancer cell proliferation, migration, invasion, and angiogenesis via miR‐378/Smad4. Further, circATRNL1 inhibited ovarian cancer abdominal metastasis *in vivo* through miR‐378/Smad4, and low level of circATRNL1 was associated well with low survival rate of ovarian cancer. Together, our study reveals an essential role of circATRNL1/miR‐378/Smad4 in ovarian cancer and sheds light on the molecular mechanisms underlying ovarian cancer development.

## 2 Materials and methods

### 2.1 Human ovarian cancer samples

Human ovarian cancer tissues and their matched adjacent noncancer ovarian tissues were obtained from 56 ovarian cancer patients during surgery from the First Hospital Affiliated Soochow University. None preoperative treatments were given to the patients. All patients have been fully informed of the study and consented, and the study has been approved by the ethics committee of the First Hospital Affiliated Soochow University. Ovarian cancer and noncancer tissues were quick‐frozen in liquid nitrogen right after and stored at −80 °C for further experiments. The study methodologies conformed to the standards set by the Declaration of Helsinki.

### 2.2 Cell culture and transfection

Four human ovarian cancer cell lines (A2780, SKOV3, CAOV‐3, and SNU119) and one normal human ovarian surface epithelial cell line (IOSE80) were obtained from the Cell Bank of the Chinese Academy of Sciences (Shanghai, China). All cells were grown in the Dulbecco's modified Eagle's medium (DMEM) (Gibco, Las Vagas, NV, USA) with supplements of 10% fetal bovine serum (FBS; ThermoFisher Scientific, Waltham, MA, USA) and 1% antibiotics penicillin/streptomycin and cultured in the standard incubator with 95% humidity and 5% CO_2_ at 37 °C. Human umbilical vein endothelial cells (HUVECs) were obtained from ATCC (Manassas, VA, USA) and were cultured in endothelial cell growth medium (Sigma‐Aldrich, Burlington, MA, USA).

The circATRNL1 full length was cloned into the overexpression plasmid. miR‐378 mimics and inhibitor were purchased from Genepharma (Shanghai, China). Sh‐Smad4 and control shRNA were synthesized from Genepharma. Cell transfection was carried out by using Lipofectamine 3000 (Invitrogen, Carlsbad， CA, USA) based on the manufacturer's instruction. In brief, cells were grown up to 80% confluence and 1–2 μg plasmid together with 1–2 μL Lipofectamine 3000 (m/v, 1 : 1) were added into the culture medium. Cells were harvested for further analysis 48 h after transfection. Stable cell lines were screened and picked by using appropriate antibiotics (puromycin, 3 μg·μL^−1^, Sigma, Burlington, MA, USA) for 7–10 days following virus infection.

### 2.3 Fluorescence *in situ* hybridization

RNA fluorescence *in situ* hybridization (FISH) was performed using the Stellaris kit (Biosearch Technologies, Petaluma, CA, USA). Cells were washed with PBS, fixed with 4% formaldehyde for 10–15 min at room temperature, rewashed with PBS, and then incubated in 70% ethanol overnight at 4 °C for permeabilization. Cells were again washed with PBS and incubated with specific probes diluted in hybridization buffer overnight at 37 °C in the dark, followed by three times washes with wash buffer. DAPI was added to stain the nucleus. Cells were mounted using the mounting medium and imaged with confocal microscopy. Probes used were generated and purchased from Stellaris: Cyst‐labeled circATRNL1 (Biosearch, Petaluma, CA, USA).

### 2.4 Colony formation assay

Transfected cells were plated in the 12‐well culture plates and grown in the incubator for 1 week. Culture medium was removed, and cells were fixed with 10% methanol for 10–15 min at room temperature. Afterward, cells were incubated with 1% crystal violet for 10 min followed by imaging with EVOS FL Imaging System (Life Technologies, New York, NY, USA). The number of colonies was analyzed with imagej (NIH, Bethesda, MD, USA).

### 2.5 Cell apoptosis assay

Annexin‐V‐FITC apoptosis Detection Kit (Sigma‐Aldrich) was used to measure cell apoptosis. Transfected cells were washed with regular PBS first and then harvested with 1× binding buffer. Five microlitre of Annexin‐V‐FITC and 5 μL of propidium iodide (PI) were added into the cell suspension for 15 min for staining. Afterward, cell suspensions were analyzed using the flow cytometer (Bio‐Rad, Berkeley, CA, USA).

### 2.6 Transwell assay

Transfected cells were cultured in the serum‐free DMEM medium on the upper chamber of transwell filter chamber filters that was precoated with Matrigel (Sigma‐Aldrich). DMEM medium containing 10% FBS was put in the lower chamber. Cells were grown in the incubator for 24 h. Afterward, the upper chamber was discarded while cells that migrated to the lower chamber were fixed in 4% paraformaldehyde for 10–15 min at room temperature. 0.2% crystal violet was added to incubate with the cells and then the stained cells were analyzed. To analyze the invasion ability of cancer cells, the upper chamber was precoated with extracellular matrix (Sigma‐Aldrich). Twenty‐four hours later, cells growing in the lower chamber were analyzed with same procedures.

### 2.7 Tube formation assay

5 × 10^4^ HUVECs were plated and co‐cultured with the same amount of transfected ovarian cancer cells (SKOV3 and CAOV3) on the plate that was precoated with Matrigel Basement Membrane Matrix (Sigma‐Aldrich). After 6 h's incubation, cells were fixed with 4% PFA for 10–15 min at room temperature followed by PBS wash. The tube formation ability of HUVECs was measured with a light microscopy.

### 2.8 RNA immunoprecipitation (RIP) assay

Transfected cells were homogenated in the lysis buffer (150 mm NaCl, 50 mm Tris/HCl, 2 mm EDTA, 0.5% sodium deoxycholate, 1% NP‐40) containing RNase inhibitors and protease inhibitor cocktail (Abcam, Burlington, MA, USA). The extracted protein was incubated with specific antibodies (anti‐Ago2, 1 : 1000 dilution; or IgG antibody as control) (Abcam) overnight at 4 °C. On the next day, the mixture was pulled down by using protein G Sepharose 4 beads (Abcam). The beads coupled with the extraction were washed with wash buffer and then digested with proteinase K (Sigma‐Aldrich) for 1.5 h. The elution was collected to isolate RNA with the Trizol reagent (Invitrogen). Quantitative RT‐PCR was performed to measure the RNA yield and the primers used were listed in the qRT‐PCR section.

### 2.9 Dual‐luciferase report assay

The Phusion Mutagenesis kit (ThermoFisher, Waltham, MA, USA) was used to mutate the binding sites according to the manufactures' instructions. cDNAs that included the wild‐type sequences or mutated binding sequences with miR‐378 in circATRNL1 and Smad4 3′ UTR were cloned into the psiCHECK luciferase reporter vector (Promega, Madison, WI, USA). Ovarian cancer cells were co‐transfected with the recombinant plasmid together with miR‐378 mimics or NC. Twenty‐four hours after the co‐transfected cells were harvested in the Reporter Lysis Buffer, the luciferase activity of each sample was measured using the Dual‐Luciferase Reporter Assay System (Promega).

### 2.10 Nude mice intraperitoneal xenograft

The nude mice (6 weeks) were purchased from SJA Laboratory Animal Co., Ltd (Hunan, China; *n* = 20) and kept in the standard animal facility room. All animal experiments have been approved by the Animal Care and Use Committee of the First Hospital Affiliated Soochow University. The suspended circATRNL1‐transfected human ovarian cancer cells (5 × 10^6^) were injected into the abdominal cavities of nude mice to induce tumors. The number of tumor nudes was counted after 30 days, and tumor weight was measured as well. Tumor tissues were harvested for further immunohistochemistry or biochemical analysis.

### 2.11 Immunohistochemistry

Ovarian cancer tissues were fixed in 4% formalin, embedded in paraffin, and then sliced into 20‐μm‐thick sections. The paraffin sections were dried for 45 min at 60 °C, deparaffinized with xylene, and rehydrated by wash with a graded concentration of alcohol (100–80%). The tissue sections were blocked with 10% BSA for 2 h at room temperature and then incubated with specific primary antibodies overnight at 4 °C. On the next day, slices were washed with PBS three times followed by incubation with specific secondary antibodies for 2 h at room temperature. Signals were analyzed with HRP IHC detection kit (Abcam) according to the manufacturer's instructions. Primary antibodies used in this study are as follows: rabbit polyclonal anti‐Smad4 antibody (1 : 500; Cell Signaling, Boston, MA, USA) and rabbit polyclonal anti‐CD31 antibody (1 : 500; Abcam, Burlington, MA, USA).

### 2.12 RNA extraction and qRT‐PCR

Trizol reagent (Invitrogen, Carlsbad, CA, USA) was used to isolate total RNAs from tissues or cultured cells according to the manufacturer's instructions. DNaseI was included into the lysis buffer to avoid the contamination of DNA. 1–2 μg total RNA of each sample was used for reverse transcription and then amplified by PCR with standard kits (Invitrogen, MO, USA). Relative expression levels of circRNA, miRNA, or mRNA were normalized to 18S RNA, U6, and GAPDH, respectively, as internal controls. The following primers were used:

circATRNL1 forward primer: 5′‐ACTGGTTTCAACATTTTCTATTCAA‐3′;
circATRNL1 reverse primer: 5′‐GCTTCACCCTTCCAGTATTT‐3′;
miR‐378 forward primer: 5′‐GCGCACTGGACTTGGAGTC‐3′;
miR‐378 reverse primer: 5′‐GCAGGGTCCGAGGTATTC‐3′;
Smad4 forward primer: 5′‐CCTGTTCACAATGAGCTTGCAT‐3;
Smad4 reverse primer: 5′‐CCTACCTGAACATCCATTTCAA‐3′;
18S RNA forward primer: 5′‐CAGGATTGACAGATTGATAGC‐3′;
18S RNA reverse primer: 5′‐GAGTCTCGTTCGT TATCGGAA‐3′;
U6 forward primer: 5′‐CTCGCTTCGGCAGCACA‐3′;
U6 reverse primer: 5′‐AACGCTTCACGAATTTGCGT‐3′;
GAPDH forward primer: 5′‐GAGTCAACGGATTTGGTCGTT‐3′;
GAPDH reverse primer: 5′‐TTGATTTTGGAGGGATCTCG‐3′;

### 2.13 Western blotting

RIPA lysis buffer (ThermoFisher, Waltham, MA, USA) was utilized to extract proteins from tissues or cells according to standard protocol. Protein concentration of each sample was measured by using Pierce™ BCA Protein Assay Kit (ThermoFisher). Equal amounts of protein were loaded into SDS/polyacrylamide gels and separated through electrophoresis. Later, the proteins were transferred from the gels to PVDF membranes (Sigma‐Aldrich). The membranes were blocked with 3% BSA for half an hour at room temperature and then incubated with primary antibodies overnight at 4 °C. On the next day, the membranes were washed with TBST three times before incubation with specific secondary antibodies for 1 h at room temperature. Signals were detected by using the standard ECL kit. Primary antibodies used in the study were as follows: anti‐c‐Myc (1 : 2000; Santa Cruz, Dallas, TX, USA); anti‐Cyclin D1 (1 : 2000; Santa Cruz); anti‐MMP2 (1 : 500; Santa Cruz); anti‐MMP9 (1 : 2000; Abcam); anti‐Smad4 (1 : 2000; Cell Signaling); anti‐p‐AKT (1 : 500; Cell Signaling); anti‐AKT (1 : 2000; Cell Signaling); anti‐GAPDH (1 : 5000; Abcam).

### 2.14 Statistical analysis

All experiments were performed at least in triplicate, and the data were analyzed in graphpad prism 7 (IBM, Armonk, NY, USA). All patients with ovarian cancer were divided into either the circATRNL1‐low (*n* = 28) or circATRNL1‐high (*n* = 28) groups according to the median value of circATRNL1 expression in ovarian cancer tissues. The correlation between circATRNL1 expression and clinicopathological characteristics of ovarian cancer patients was assessed by the chi‐squared test. Kaplan–Meier method was used to calculate survival curves, and the significance was analyzed by log‐rank test. Statistical values were calculated by Student *t*‐test (unpaired two‐tailed) for two groups or one‐way ANOVA for groups more than two. Significance was determined based on the *P* values: *P < 0.05, **P < 0.01, ***P < 0.001. Error bars indicated ± SD (standard deviation).

## 3 Results

### 3.1 circATRNL1 suppressed ovarian cancer cell proliferation, migration, invasion, and angiogenesis

**Fig. 1:**
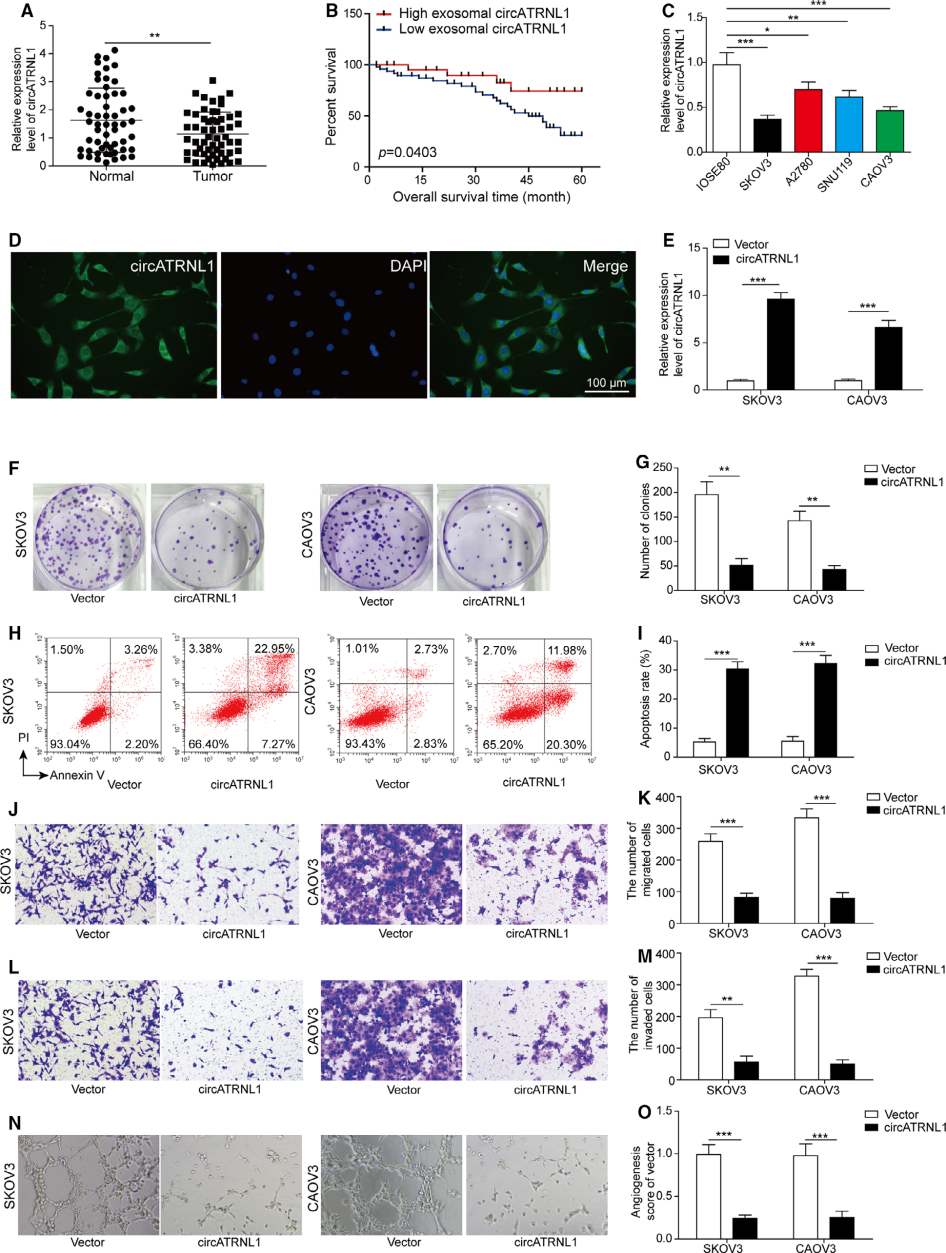
circATRNL1 suppressed ovarian cancer cell proliferation, migration, invasion, and angiogenesis. (A) qRT‐PCR analysis to measure circATRNL1 level in ovarian cancer tissues from ovarian cancer patients. Relative expression level was normalized to 18S RNA and GAPDH. *n* = 56. (B) Survival rates of ovarian cancer patients with different levels of circATRNL1. (C) qRT‐PCR analysis to measure circATRNL1 level in ovarian cancer cells. Relative expression level was normalized to 18S RNA and GAPDH. (D) FISH analysis of localization of circATRNL1 in IOSE80 cells. Scale bar: 100 μm. (E) qRT‐PCR analysis of circATRNL1 level inSKOV3 and CAOV3 cells transfected with circATRNL1 overexpressing vector. Relative expression level was normalized to 18S RNA and GAPDH. (F, G) Colony formation assay was used to measure proliferation of transfected cancer cells. (H, I) Flow cytometry was used to analyze the apoptosis percentage of transfected cancer cells. (J–M) Transwell assay was performed to assess the migration and invasion of transfected cancer cells. (N, O) Tube formation analysis of angiogenesis. All the results were shown as mean ± SD (*n* = 3), which were three separate experiments performed in triplicate. *P < 0.05, **P < 0.01, and ***P < 0.001.

To study the role of circATRNL1 in ovarian cancer, we first examined the expression of circATRNL1 in ovarian cancer tissues and cells. We obtained ovarian cancer tissues from ovarian cancer patients and found that circATRNL1 was significantly reduced in the cancer tissues compared to adjacent nontumor tissues (Fig. 1A). We then examined the correlation between circATRNL1 level and survival rate of ovarian cancer patients. We found that patients with higher level of circATRNL1 had longer survival time than patients with lower level of circATRNL1 (Fig. 1B). As shown in Table 1, there were significantly more patients from the low circATRNL1 level group diagnosed at advanced FIGO stages and exhibiting lymph node metastasis than from the high circATRNL1 group. Together, these results indicate that circATRNL1 level is reduced in ovarian cancer patients and that the low level is correlated with poor survival rate.

**Table 1:** Correlation of the expression levels of circATRNL1 with the clinicopathological characteristics of ovarian cancer patients. FIGO, Federation International of Gynecology and Obstetrics; G1, well differentiated; G2, moderately differentiated; G3, poorly differentiated.

Clinical parameters	Cases (*n*)	circATRNL1 expression		*P*‐value (*P < 0.05)
		High (*n*)	Low (*n*)	
Age (years)				
< 55	24	13	11	0.589
≥ 55	32	15	17	
Histological subtype				
Serous	30	14	16	0.592
Other	26	14	12	
Tumor size (cm)				
< 8	23	15	8	0.057
≥ 8	33	12	20	
FIGO stage				
I–II	26	17	9	0.032*
III–IV	30	11	19	
Lymph node metastasis				
Yes	23	7	16	0.015*
No	33	21	12	
Histological grade				
G1	29	16	13	0.422
G2 + G3	27	12	15	

To further study the function, we used four different ovarian cancer cell lines and consistently observed that circATRNL1 level in ovarian cancer cells was greatly lower than that in normal human ovarian surface epithelial cell (Fig. 1C). The level of circATRNL1 was lowest in SKOV3 and CAOV3 cells, and therefore, we used these two cell lines for subsequent experiments (Fig. 1C). With FISH experiment, we showed that circATRNL1 was primarily localized in cytoplasm of human ovarian surface epithelial cell IOSE80 (Fig. 1D). Transfection of circATRNL1 overexpression vector in cells greatly increased the level of circATRNL1 (Fig. 1E). With colony formation assay, we found that overexpression of circATRNL1 significantly decreased the number for colonies formed (Fig. 1F,G). Consistently, using flow cytometry, we found that overexpression of circATRNL1 greatly enhanced the percentage of cancer cell apoptosis (Fig. 1H,I). Using transwell assay, we showed that circATRNL1 also decreased the number of migration and invasion cells (Fig. 1J,K). With tube formation assay, we observed that overexpression of circATRNL1 greatly decreased the number of branches (Fig. 1N,O). Taken together, these results indicate that circATRNL1 is downregulated in ovarian cancer and that overexpression of circATRNL1 promotes cell apoptosis and inhibits cancer cell proliferation, migration, invasion, and angiogenesis.

### 3.2 circATRNL1 directly bound to miR‐378 in ovarian cancer cells

**Fig. 2:**
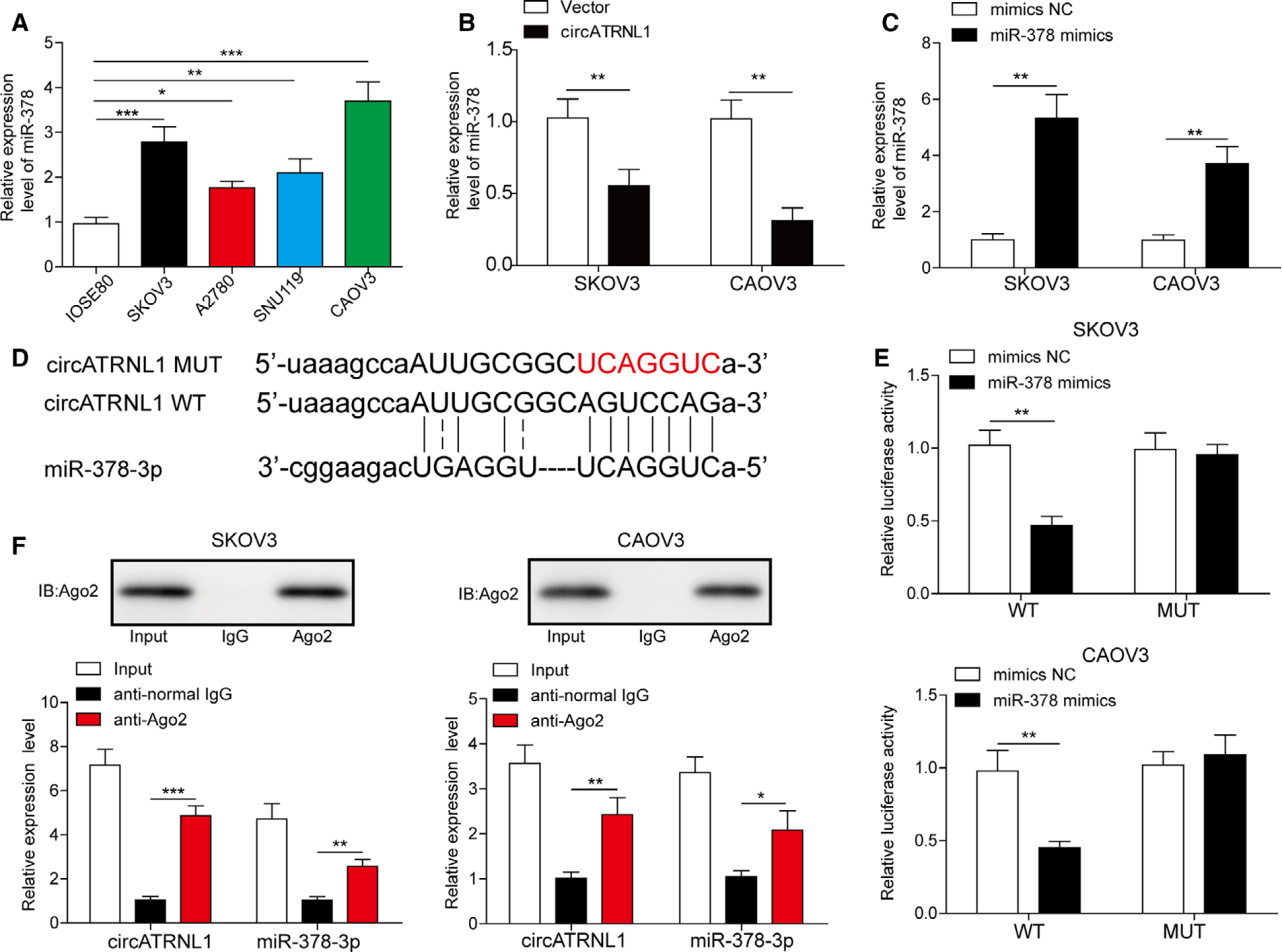
circATRNL1 directly bound to miR‐378. (A) qRT‐PCR analysis to measure miR‐378 levels in ovarian cancer cells. (B) qRT‐PCR analysis of miR‐378 level in cells transfected with circATRNL1 or vector. (C) qRT‐PCR analysis of miR‐378 level in transfected cells. Relative expression level of miR‐378 was normalized to U6. (D) The binding sites between circATRNL1 and miR‐378. (E) Relative luciferase activity of WT‐circATRNL1 and MUT‐circATRNL1 in cells transfected with mimics NC, miR‐378 mimics. (F) Ago2 antibody pulled down more circATRNL1 and miR‐378 compared to IgG control. All the results were shown as mean ± SD (*n* = 3), which were three separate experiments performed in triplicate. *P < 0.05, **P < 0.01, and ***P < 0.001.

circRNAs have been shown to function as a sponge for microRNAs [8]. We next investigated whether circATRNL1 bound to any microRNA and we focused on miR‐378. In contrast to the change of circATRNL1, miR‐378 level was significantly elevated in ovarian cancer cells compared to normal human ovarian surface epithelial cell (Fig. 2A). Moreover, cells overexpressed with circATRNL1 had much lower level of miR‐378 than cells transfected with vector control (Fig. 2B), suggesting that circATRNL1 negatively regulates miR‐378 expression. As expected, cells transfected with miR‐378 mimics had higher levels of miR‐378 than cells transfected with mimics NC (Fig. 2C). Using the Starbase database, we found some complementary binding sites between circATRNL1 and miR‐378 (Fig. 2D). To validate the interactions, we performed dual‐luciferase assay. miR‐378 mimics greatly decreased the luciferase activity of WT‐circATRNL1 but not the MUT‐circATRNL1 where the predicted binding sites were mutated (Fig. 2E). Moreover, using Ago2‐RIP assay, we showed that immunoprecipitation with Ago2 antibody greatly pulled down both circATRNL1 and miR‐378 in ovarian cancer cells compared with immunoprecipitation with normal IgG antibody (Fig. 2F). Together with these data, we demonstrate that circATRNL1 directly binds with miR‐378.

### 3.3 circATRNL1 inhibited cancer cell proliferation, migration, invasion, and angiogenesis via miR‐378

**Fig. 3:**
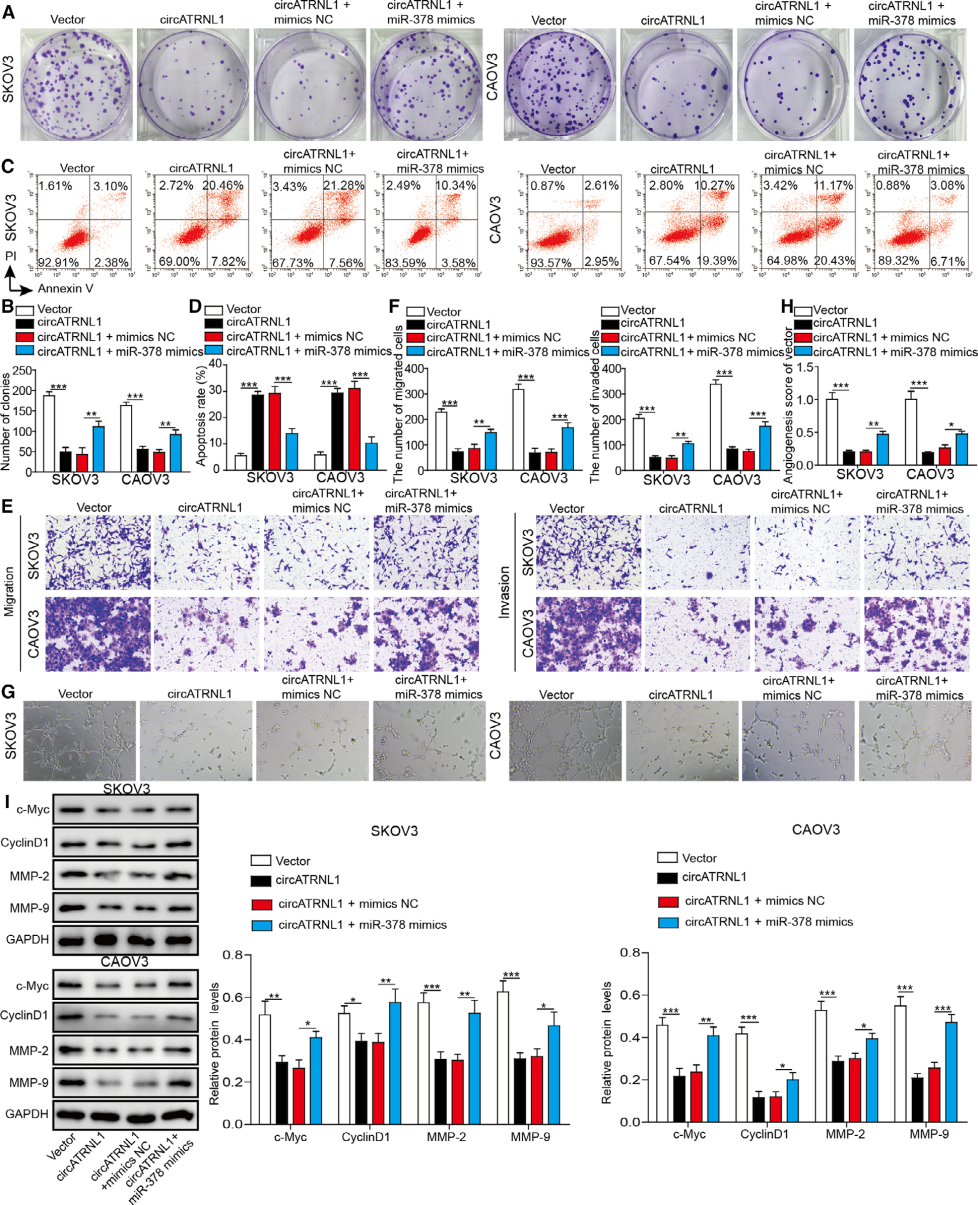
circATRNL1 inhibited cancer cell proliferation, migration, invasion, and angiogenesis via miR‐378. (A, B) Colony formation assay was carried out to measure proliferation of transfected cancer cells. (C, D) Flow cytometry was performed to determine the apoptosis percentage of transfected cancer cells. (E, F) Transwell assay was employed to determine the migration and invasion abilities of transfected cancer cells. (G, H) Tube formation analysis of angiogenesis of transfected cells. (I) Western blotting analysis of protein levels of c‐Myc, Cyclin D1, PKM2, MMP‐2, and MMP‐9. The protein levels were normalized to GAPDH. All the results were shown as mean ± SD (*n* = 3), which were three separate experiments performed in triplicate. *P < 0.05, **P < 0.01, and ***P < 0.001.

We then examined how the interaction of circATRNL1 and miR‐378 was involved in ovarian cancer. Overexpression of circATRNL1 decreased the number of colonies formed in cancer cells. However, co‐overexpression of miR‐378 rescued the number of colonies (Fig. 3A,B). In addition, co‐overexpression of miR‐378 decreased the percentage of cell apoptosis induced by circATRNL1 overexpression (Fig. 3C,D). Similarly, using transwell assay, we found that the reduced migration and invasion abilities of cancer cells induced by circATRNL1 were recovered by co‐expression of miR‐378 (Fig. 3E,F). The angiogenesis was rescued as well by miR‐378 as miR‐378 mimics increased the number of branches (Fig. 3G,H). At the molecular level, we found that circATRNL1 significantly reduced the levels of proliferation‐related proteins (c‐Myc and Cyclin D1) and migration‐related proteins (MMP‐2 and MMP‐9) while co‐overexpression of miR‐378 recovered the levels of those proteins (Fig. 3I). Altogether, our results demonstrate that circATRNL1 promotes cancer cell apoptosis and inhibits cell proliferation, migration, invasion, and angiogenesis via targeting miR‐378.

### 3.4 miR‐378 activated AKT signaling via targeting Smad4

**Fig. 4:**
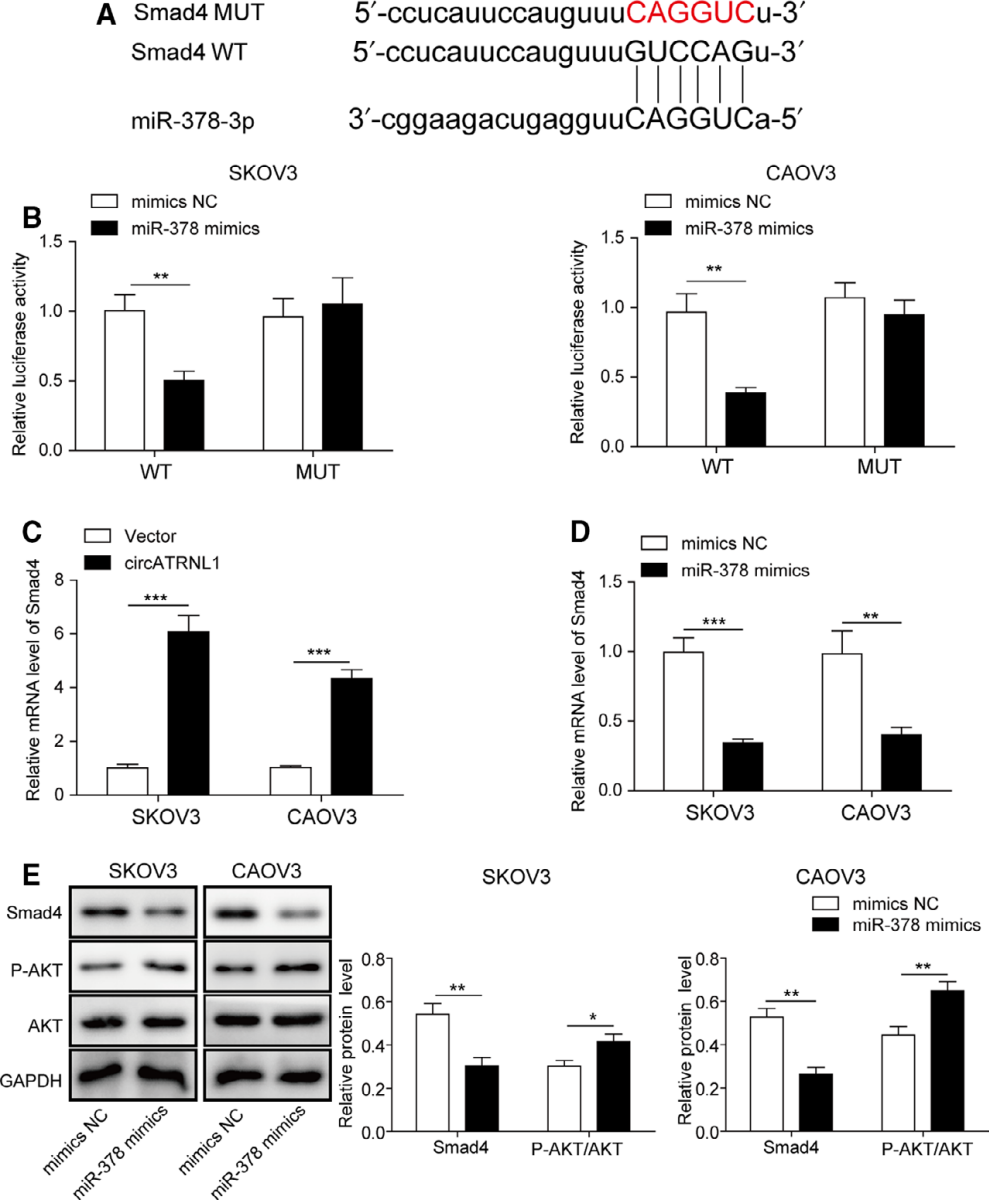
miR‐378 activated AKT signaling via targeting Smad4. (A) The predicted binding sites between miR‐378 and Smad4 mRNA. (B) Relative luciferase activity of WT‐Smad4 and MUT‐Smad4 in cells transfected with mimics NC, miR‐485‐3p mimics. (C) qRT‐PCR analysis to measure Smad4 mRNA levels in SKOV3 and CAOV3 cells transfected with circATRNL1 overexpressing vector. Relative mRNA level was normalized to GAPDH. (D) qRT‐PCR analysis to measure Smad4 mRNA levels in SKOV3 and CAOV3 cells transfected with miR‐378 mimics. Relative mRNA level was normalized to GAPDH. (E) Western blot analysis of protein levels of Smad4, p‐AKT, and AKT. The protein levels were normalized to GAPDH. All the results were shown as mean ± SD (*n* = 3), which were three separate experiments performed in triplicate. *P < 0.05, **P < 0.01, and ***P < 0.001.

microRNAs usually function to repress expressions of target genes by binding to their complementary mRNAs [13]. With bioinformatic analysis (Starbase: http://starbase.sysu.edu.cn/index.php), we detected some complementary binding sites between miR‐378 and Smad4 (Fig. 4A). We confirmed this binding via dual‐luciferase assay. miR‐378 mimics significantly reduced the luciferase activity of WT‐Smad4 but not the MUT‐Smad4, where the predicted binding sites with miR‐378 were mutated (Fig. 4B). Overexpression of circATRNL1 could upregulate Smad4 mRNA level (Fig. 4C). Moreover, overexpression of miR‐378 greatly decreased Smad4 mRNA level in cancer cells (Fig. 4D). These results suggest that miR‐378 represses the expression of Smad4 and that circATRNL1 disinhibits that regulation via binding to miR‐378. Smad4 has been shown implicated in AKT signaling. We thus investigated how miR‐378 affected AKT signaling. As expected, overexpression of miR‐378 reduced Smad4 protein level (Fig. 4E). However, miR‐378 mimics increased AKT phosphorylation level, suggesting that miR‐378 activates AKT signaling by targeting Smad4 (Fig. 4E).

### 3.5 Knockdown miR‐378 inhibited cancer cell proliferation, migration, invasion, and angiogenesis via Smad4

**Fig. 5:**
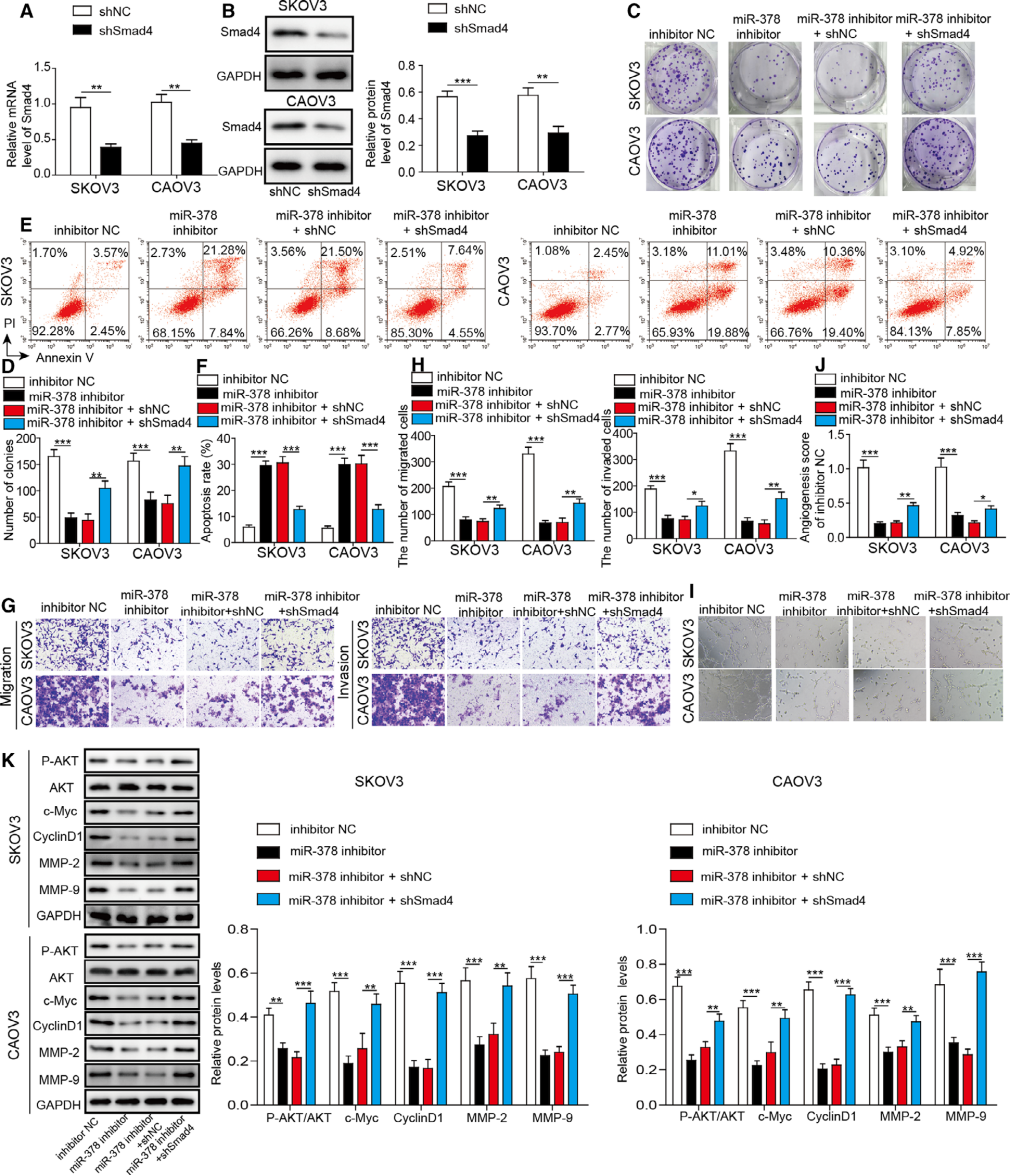
Knockdown miR‐378 inhibited cancer cell proliferation, migration, invasion, and angiogenesis via Smad4. (A) qRT‐PCR was used to analyze Smad4 mRNA level in SKOV3 and CAOV3 cells transfected with shSmad4. Relative mRNA level was normalized to GAPDH. (B) Western blotting to analyze Smad4 protein level in transfected cells. The protein levels were normalized to GAPDH. (C, D) Colony formation assay was used to measure proliferation of transfected cancer cells. (E, F) Flow cytometry was performed to analyze the apoptosis percentage of transfected cancer cells. (G, H) Transwell assay to determine the migration and invasion of transfected cancer cells. (I, J) Tube formation analysis of angiogenesis of transfected cells. (K) Western blotting was performed to measure protein levels of p‐AKT, AKT, c‐Myc, Cyclin D1, PKM2, MMP‐2, and MMP‐9. The protein levels were normalized to GAPDH. All the results were shown as mean ± SD (*n* = 3), which were three separate experiments performed in triplicate. *P < 0.05, **P < 0.01, and ***P < 0.001.

We next investigated the role of miR‐378/Smad4 axis in ovarian cancer. Cells transfected with sh‐Smad4 had much lower levels of Smad4 mRNA and protein compared to cells transfected with sh‐NC (Fig. 5A,B). Knockdown of miR‐378 through miR‐378 inhibitor decreased the colony number (Fig. 5C,D). Nevertheless, co‐transfection with sh‐Smad4 rescued the number of colonies formed compared with cells transfected with miR‐378 inhibitor alone (Fig. 5C,D). Similarly, with flow cytometry, we showed that miR‐378 inhibitor increased cell apoptosis percentage, but co‐expression of sh‐Smad4 decreased the percentage (Fig. 5E,F). With the transwell assay, we observed that miR‐378 inhibitor significantly diminished the number of migration and invasion cells while co‐transfection with sh‐Smad4 recovered the numbers (Fig. 5G,H). In tube formation assay, the number of branches was decreased in cells transfected with miR‐378 inhibitor, but sh‐Smad4 reversed the effect of miR‐378 inhibitor (Fig. 5I,G). At the molecular level, we showed that miR‐378 inhibitor greatly decreased p‐AKT level, as well as the levels of proliferation‐related proteins (c‐Myc and Cyclin D1) and migration‐related proteins (MMP‐2 and MMP‐9) (Fig. 5K). Co‐expression of sh‐Smad4 restored the levels of all those proteins including p‐AKT, proliferation‐related proteins, and migration‐related proteins (Fig. 5K). Taken together, these results provide evidence that inhibitor of miR‐378 suppresses cancer cell proliferation, migration, invasion, and angiogenesis through Smad4.

### 3.6 Knockdown of Smad4 mediated the inhibition of cancer cell proliferation, migration, invasion, and angiogenesis induced by circATRNL1

**Fig. 6:**
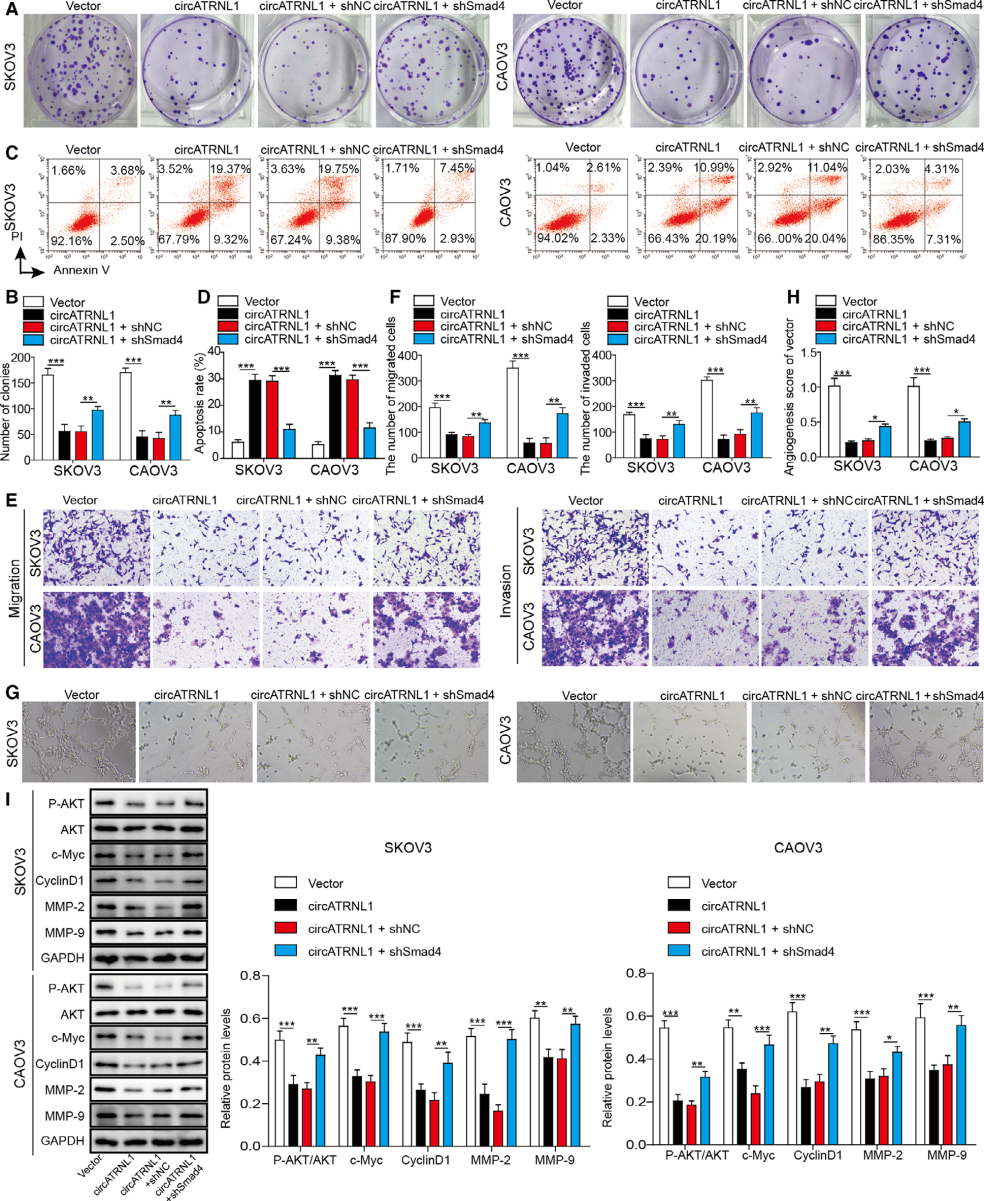
Smad4 mediated the inhibition of cancer cell proliferation, migration, invasion, and angiogenesis by circATRNL1. (A, B) Colony formation assay was used to determine the proliferation of transfected cancer cells. (C, D) Flow cytometry was performed to assess the apoptosis percentage of transfected cancer cells. (E, F) Transwell assay was used to determine the migration and invasion of transfected cancer cells. (G, H) Tube formation analysis of angiogenesis of transfected cells. (I) Western blotting was performed to measure protein levels of p‐AKT, AKT, c‐Myc, Cyclin D1, PKM2, MMP‐2, and MMP‐9. The protein levels were normalized to GAPDH. All the results were shown as mean ± SD (*n* = 3), which were three separate experiments performed in triplicate. *P < 0.05, **P < 0.01, and ***P < 0.001.

We have showed that circATRNL1 regulated ovarian cancer cell proliferation and migration via binding to miR‐378 and that miR‐378 modulated cancer cell proliferation and migration through targeting Smad4. To further confirm the function of circATRNL1/miR‐378/Smad4 axis in ovarian cancer, we next studied how Smad4 affected the function of circATRNL1 in ovarian cancer. Consistent with results aforementioned, overexpression of circATRNL1 decreased the number of colonies formed and increased the cell apoptosis percentage (Fig. 6A–D). Also circATRNL1 reduced the migration and invasion of cancer cells, as well as angiogenesis (Fig. 6E–H). Co‐expression of sh‐Smad4 reversed all the effects induced by circATRNL1 (Fig. 6A–H). Moreover, circATRNL1 diminished the levels of p‐AKT, proliferation‐related proteins (c‐Myc and Cyclin D1), and migration proteins (MMP‐2 and MMP‐9) while co‐expression of sh‐Smad4 restored the expression of those proteins (Fig. 6I). We, therefore, conclude that circATRNL1 regulates ovarian cancer cell proliferation, migration, invasion, and angiogenesis through Smad4.

### 3.7 Overexpression of circATRNL1 inhibited ovarian cancer abdominal metastasis via miR‐378/Smad4 *in vivo*

**Fig. 7:**
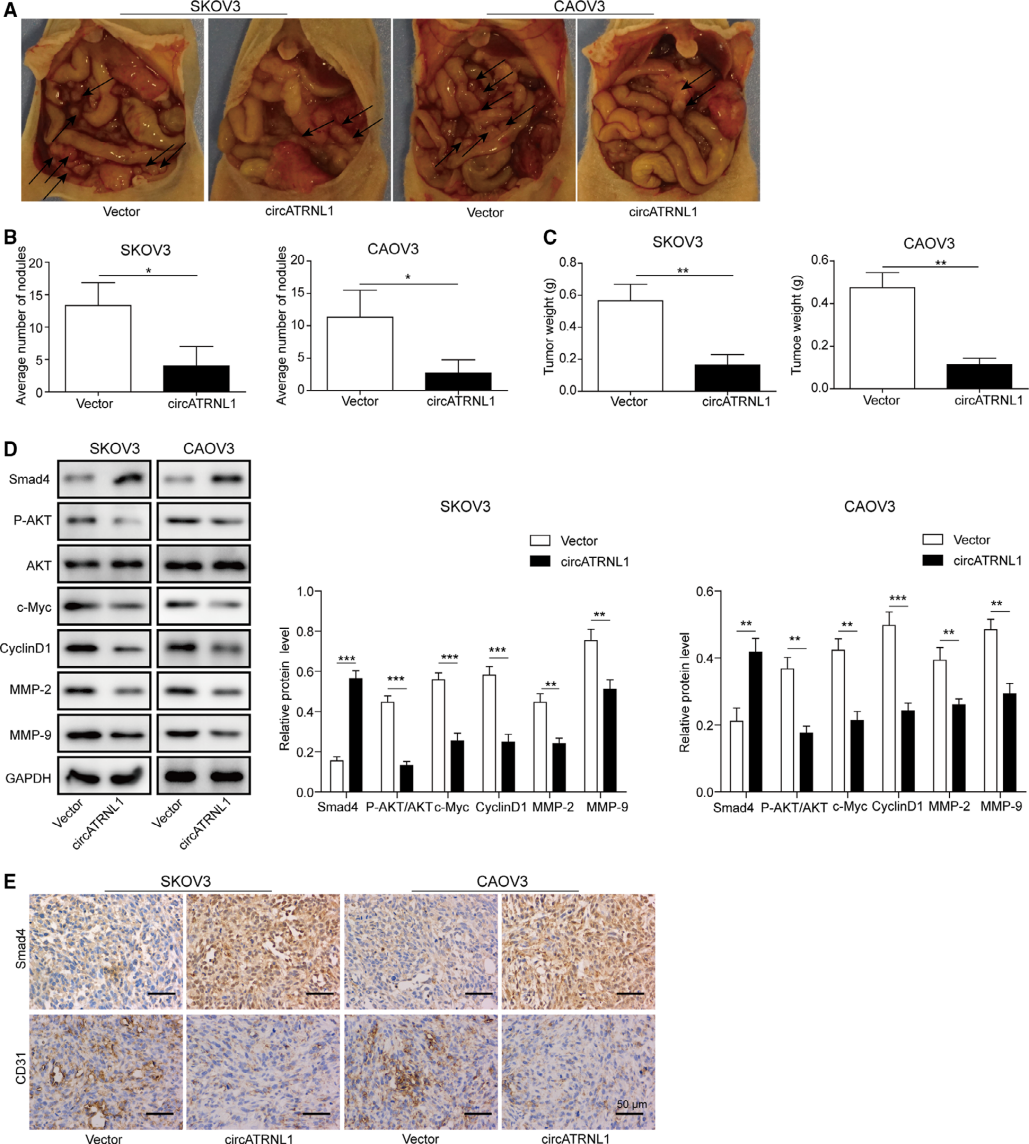
Overexpression of circATRNL1 inhibited ovarian cancer abdominal metastasis via miR‐378/Smad4 *in vivo*. (A) Representative images of tumor in mice‐bearing SKOV3 and CAOV3 cells transfected with vector and circATRNL1. *n* = 5. (B) Quantifications of the number of tumor nodules in the abdomen. (C) Quantification of tumor weight. (D) Western blot analysis of protein levels of Smad4, p‐AKT, AKT, c‐Myc, Cyclin D1, MMP‐2, and MMP‐9 in tumor tissues from different groups of mice. The protein levels were normalized to GAPDH. (E) Immunohistochemistry analysis of Smad4 and CD31 levels in tumor tissues from different groups of mice Scale bar: 50 μm. The results were shown as mean ± SD (*n* = 3), which were three separate experiments performed in triplicate. *P < 0.05, **P < 0.01, and ***P < 0.001.

We then investigated the role of circATRNL1/miR‐378/Smad4 in ovarian cancer *in vivo* using nude mice intraperitoneal xenograft. In mice that were injected with circATRNL1‐transfected SKOV3 and CAOV3 cells, both the number of tumor nudes and the tumor weight in the abdomen were significantly smaller than that in mice‐bearing control‐transfected ovarian cancer cells (Fig. 7A–C). With Western blotting, we found that overexpression of circATRNL1 increased Smad4 level but diminished the levels of p‐AKT, proliferation‐related proteins (c‐Myc and Cyclin D1), and migration‐related proteins (MMP‐2 and MMP‐9) (Fig. 7D). We also confirmed the higher expression of Smad4 in circATRNL1‐transfected tumors with immunohistochemistry (Fig. 7E). Also, we found CD31, the marker of angiogenesis, was reduced in the circ‐ATRNL1‐transfected group compared to control‐transfected group (Fig. 7E). These data demonstrate that circATRNL1 suppresses ovarian cancer growth and abdominal metastasis *in vivo*.

## 4 Discussion

**Fig. 8:**
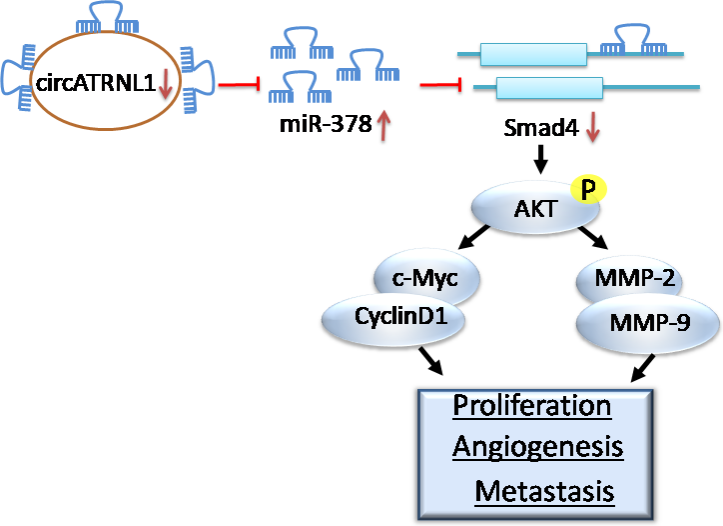
Schematic model of the role of circATRNL1/miR‐378/Smad4 axis in ovarian cancer. circATRNL1 is reduced in ovarian cancer cells, leading to an elevation of miR‐378 and subsequently a reduction of Smad4. As a result, AKT signaling is activated, promoting the cancer cell proliferation, angiogenesis, as well as metastasis.

Owing to its asymptomatic nature and absence of early detection markers, ovarian cancer is often diagnosed at late stages with high metastasis [1, 2, 3, 4]. Consequently, the prognosis of ovarian cancer is very poor and it has a very high incidence‐to‐death ratio. Understanding the pathogenesis of ovarian cancer is very necessary for better therapy or diagnosis of ovarian cancer. Here, we elucidated the function of circATRNL1/miR‐378/Smad4 in ovarian cancer. We showed that circATRNL1 was greatly reduced in ovarian cancer tissues and cells while miR‐378 was upregulated. circATRNL1 promoted ovarian cancer cell apoptosis but suppressed cancer cell proliferation, migration, and invasion, as well as angiogenesis, via targeting miR‐378/Smad4 signaling. Overexpression of circATRNL1 inhibited abdominal metastasis of ovarian cancer *in vivo* and low level of circATRNL1 was associated with low survival rate of ovarian cancer patients. These results provide strong evidence that circATRNL1 activates Smad4 signaling via miR‐378 to inhibit angiogenesis and ovarian cancer metastasis (Fig. 8).

CircRNAs constitute a novel class of noncoding RNAs whose 5′ and 3′ ends are covalently bound [6]. A huge body of studies have demonstrated that circRNAs have important functions and can modulate gene expression via competing endogenous RNAs or miRNA sponges [29, 30, 31], or interacting with RNA binding proteins [32, 33]. Moreover, emerging evidence suggests that circRNAs play a crucial role in cancer development and progression and that some circRNAs can serve as cancer biomarkers [6, 7]. They can regulate cancer cell proliferation, migration, and invasion [6]. Also circRNAs can contribute to cancer development through the regulation of angiogenesis and neovascularization [34]. For instance, circRNA MYLK promotes bladder cancer progression and metastasis via enhancing cancer cell growth and angiogenesis [35]. In the ovarian cancer, the roles of circRNAs are largely unknown. Nevertheless, several studies report dysregulated circRNAs in ovarian cancer cells, such as circRNA VPS12C‐has‐circ‐001567, circCSPP1, and circEXOC6B [36, 37, 38]. Here, we demonstrate that circATRNL1 plays a causal role in ovarian cancer. circATRNL1 was reduced in cancer tissues from ovarian cancer patients and cancer cells. Further, overexpression of circATRNL1 greatly suppressed the cancer cell proliferation, migration, and invasion, as well as angiogenesis, leading to lower metastasis *in vivo*. More importantly, we showed that low level of circATRNL1 was associated well with poor survival rate of ovarian cancer patients. These results suggest circATRNL1 may serve as a biomarker for ovarian cancer and rescuing the level of circATRNL1 could be an avenue for ovarian cancer therapy. The function of circATRNL1 is largely unknown. Besides ovarian cancer, it will be interesting to examine whether circATRNL1 has similar roles in other types of cancers, which requires future investigations. In addition, we demonstrated here that miR‐378 is the downstream target of circATRNL1. It is possible that circATRNL1 may have multiple interactors. For example, previous studies indicate that circATRNL1 sponges miR‐23a‐3p [12]. Whether similar interaction exists in ovarian cancer cells remains further studies.

Many miRNAs are implicated in cancers and miR‐378 has been shown involved in multiple cancers [15, 20, 39]. For instance, it inhibited colon cancer cell proliferation, migration, and invasion via SDAD1 [40]. However, in human lung adenocarcinoma, miR‐378 promoted cancer cell invasion by targeting RBX1 [41]. Therefore, miR‐378 has distinct roles in different types of cancers, depending on its downstream targets. In addition, miR‐378 can regulate angiogenesis, a key process for tumor progression and metastasis, by directly targeting and regulating vascular endothelial growth factor (VEGF) and other molecules [42, 43]. In ovarian cancer, the role of miR‐378 is elusive. We found miR‐378 was elevated in ovarian cancer tissues, suggesting that it functions as a tumor promoter. Indeed, we showed that knockdown of miR‐378 restrained the proliferation, migration, and invasion of ovarian cancer cells, as well as angiogenesis. Our study, together with previous studies, suggests that miR‐378 has an important role in the development of cancers. Given the important roles of miR‐378 in varieties of cancers, the upstream regulator of miR‐378, circATRNL1, might be involved as well. In addition, besides Smad4, many other downstream targets of miR‐378 have been reported, including SDAD1, SuFu, and Fus‐1 [40, 43]. Whether those targets are involved in ovarian cancers is not known and future studies are necessary to examine that.

Smad4 has a critical role in many biological processes, such as cell proliferation, migration, and development, as well as cell apoptosis [22, 24, 25]. Moreover, aberrant Smad4 expression has been observed in multiple diseases, particularly cancers [23]. Smad4 is inactivated in pancreatic duct adenocarcinoma [25, 44]. Loss of Smad4 in colorectal cancer enhanced chemoresistance to 5‐fluorouracil via activating AKT pathway [27]. Activation of Smad4 induces transcriptional changes of many genes such as c‐Myc and Cyclin D1, and these transcriptional pathways also regulate vascular remodeling and angiogenesis [45, 46, 47, 48]. Therefore, Smad4 has been implicated as a tumor suppressor [24]. In ovarian cancer, previous studies showed that deletion of Smad4 in blood vessel endothelial cells facilitates ovarian cancer metastasis [28]. However, the upstream regulators of Smad4 in this process are not well known. In the study, we showed that miR‐378 directly bound to Smad4 mRNA and circATRNL1/miR‐378 regulated ovarian cancer progression via Smad4 signaling. Our data are consistent with previous studies and indicate that loss of Smad4 promotes cancer development and progression.

## 5 Conclusions

In summary, with a combination of *in vitro* and *in vivo* methods, we show that circATRNL1 plays an essential role in ovarian cancer and functions to suppress cancer metastasis and angiogenesis via targeting miR‐378/Smad4. The important role of circATRNL1/miR‐378/Smad4 suggests that it could serve as a biomarker for ovarian cancer diagnosis or even an avenue for future therapy development.

## Conflict of interest

The authors declare no conflict of interest.

## Author contributions

JW and YL performed the research and wrote the paper. YL and XS analyzed the data. JW, YL, FRS and JHZ performed the research. All authors have read and approved the final manuscript. YGC have seen and can confirm the authenticity of the raw data.

## Ethics approval

All patients have been fully informed of the study and consented, and the study has been approved by the ethics committee of the First Hospital Affiliated Soochow University. And all animal experiments have been approved by the Animal Care and Use Committee of the First Hospital Affiliated Soochow University.
